# Supplementary material for: Plasmodium falciparum has evolved multiple mechanisms to hijack human immunoglobulin M
Source: Nat Commun. 2023 May 8;14:2650. doi: 10.1038/s41467-023-38320-z (PMC10167334; doi:10.1038/s41467-023-38320-z)
Supplement: Supplementary file 1 — Supplementary Information [file 41467_2023_38320_MOESM1_ESM.pdf]

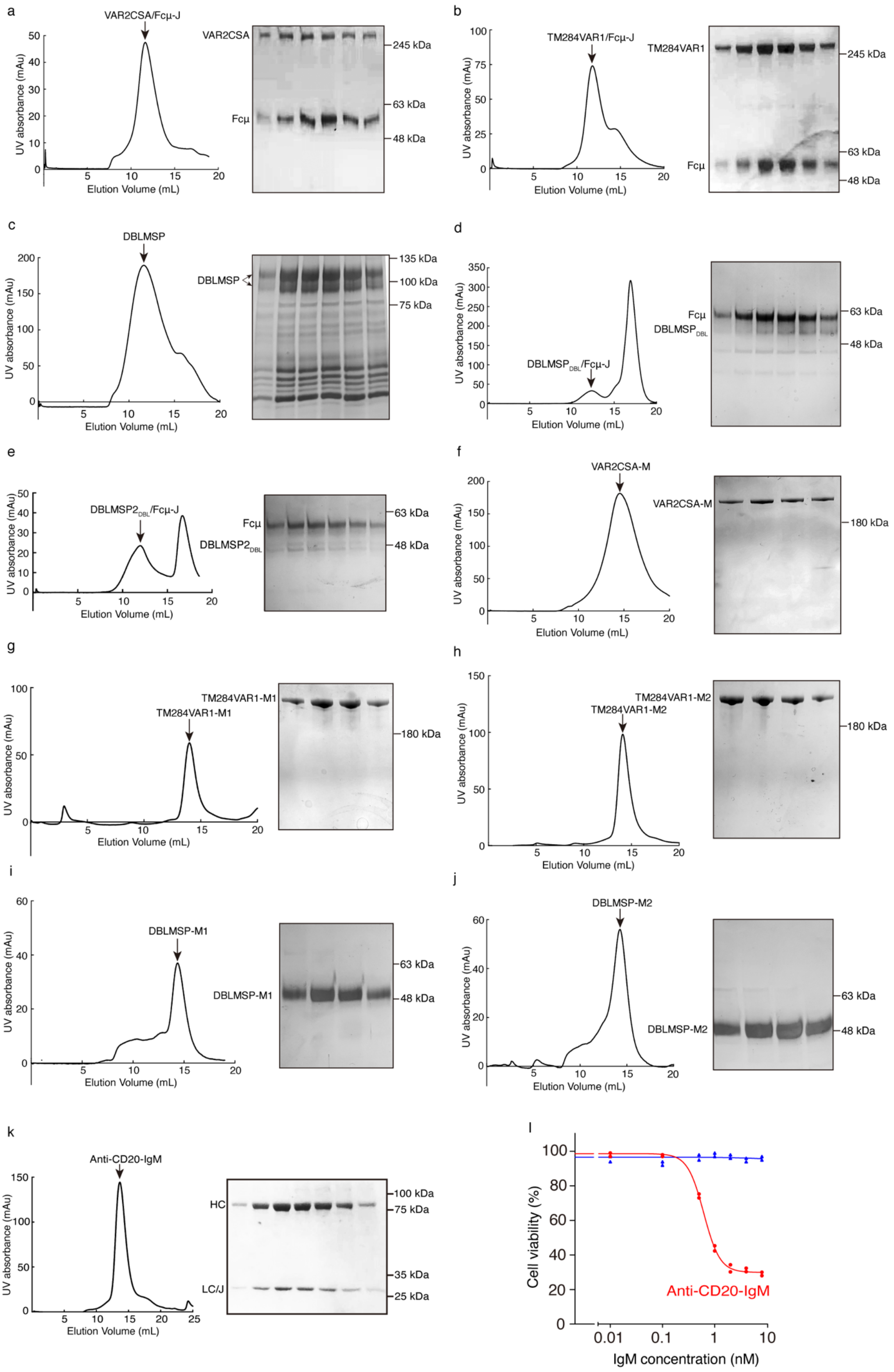
a
VAR2CSA/Fcμ-J
UV absorbance (mAu)
Elution Volume (mL)
VAR2CSA
Fcμ
245 kDa
63 kDa
48 kDa
b
TM284VAR1/Fcμ-J
TM284VAR1
Fcμ
245 kDa
63 kDa
48 kDa
c
DBLMSP
135 kDa
100 kDa
75 kDa
d
DBLMSPDBL/Fcμ-J
Fcμ
DBLMSPDBL
63 kDa
48 kDa
e
DBLMSP2DBL/Fcμ-J
Fcμ
DBLMSP2DBL
63 kDa
48 kDa
f
VAR2CSA-M
180 kDa
g
TM284VAR1-M1
180 kDa
h
TM284VAR1-M2
180 kDa
i
DBLMSP-M1
63 kDa
48 kDa
j
DBLMSP-M2
63 kDa
48 kDa
k
Anti-CD20-IgM
HC
LC/J
100 kDa
75 kDa
35 kDa
25 kDa
l
Cell viability (%)
IgM concentration (nM)
Anti-CD20-IgM

Supplementary Figure 1. Purification of the proteins and protein complexes in this study.

a. Size exclusion chromatography of the VAR2CSA–Fcμ–J complex (left) and associated SDS-PAGE (right). All the purification experiments and corresponding SDS-PAGE analyses in this paper have been repeated at least two times with similar results.

b. Size exclusion chromatography of the TM284VAR1–Fcμ–J complex and SDS-PAGE.

c. Size exclusion chromatography of full-length DBLMSP and SDS-PAGE.

d. Size exclusion chromatography of the $DBLMSP_{DBL}$–Fcμ–J complex and SDS-PAGE.

e. Size exclusion chromatography of the $DBLMSP2_{DBL}$–Fcμ–J complex and SDS-PAGE.

f. Size exclusion chromatography of the VAR2CSA-M and SDS-PAGE.

g. Size exclusion chromatography of the TM284VAR1-M1 and SDS-PAGE.

h. Size exclusion chromatography of the TM284VAR1-M2 and SDS-PAGE.

i. Size exclusion chromatography of the DBLMSP-M1 and SDS-PAGE.

j. Size exclusion chromatography of the DBLMSP-M2 and SDS-PAGE.

k. Size exclusion chromatography of anti-CD20 IgM and SDS-PAGE. HC: heavy chain; LC: light chain; J: J-chain. The light chain and the J-chain have similar molecular weights and could not be separated in SDS-PAGE.

l. Anti-CD20 IgM, but not another similarly recombinant IgM targeting the receptor binding domain of the SARS-CoV-2 spike (anti-RBD IgM), triggers complement-dependent cytotoxicity of OCI-Ly10 cells in the presence of human complement. Two technical replicates are depicted for each experiment, and the means are used to construct the plots. Source data for two representative experiments are provided in the Source Data file.

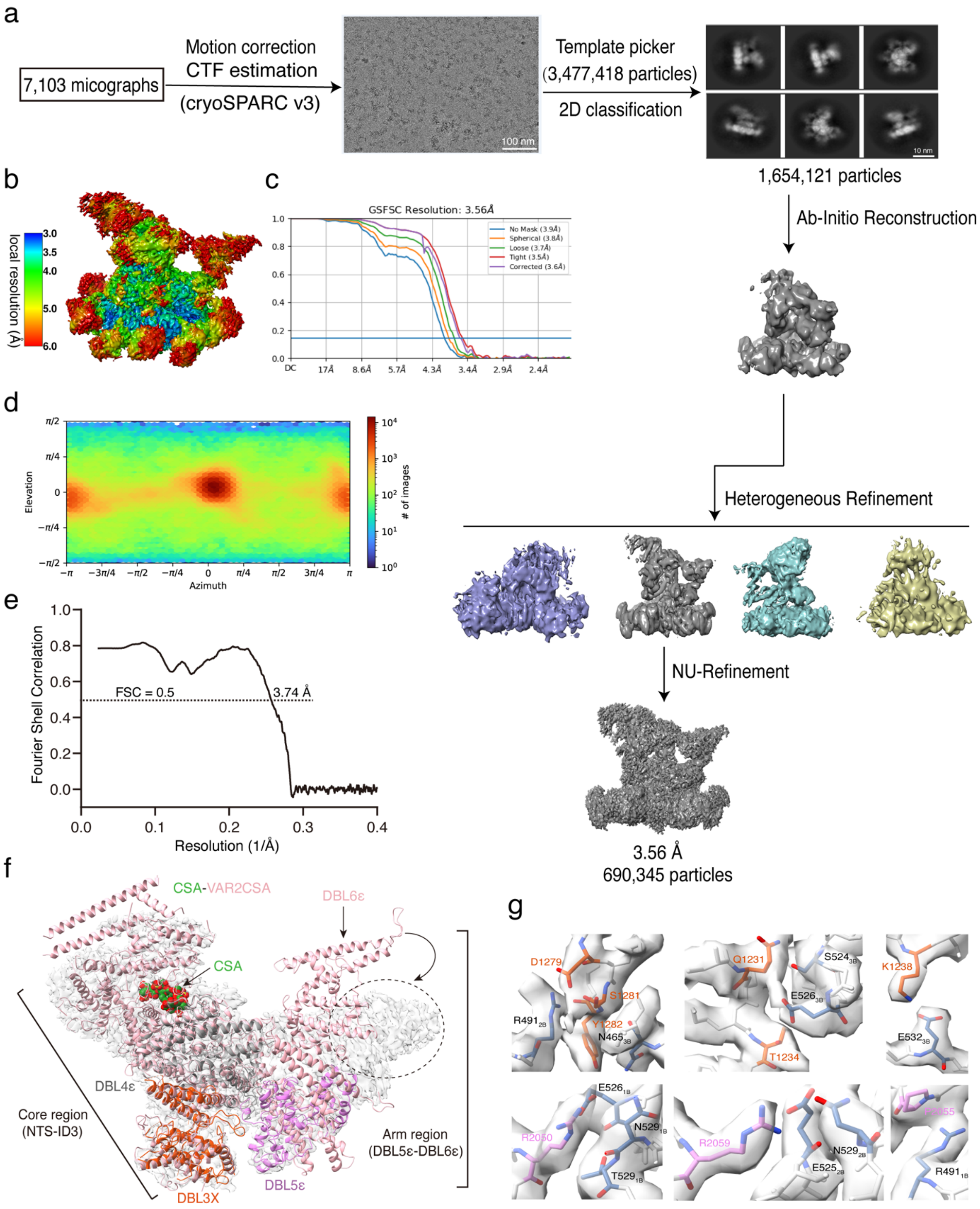

Supplementary Figure 2. Workflow for the 3D reconstruction of the VAR2CSA–Fcμ–J cryo-EM structure.

a. Flow chart for image processing of the VAR2CSA–Fcμ–J data.

b. Resolution estimations of the final map of VAR2CSA–Fcμ–J.

c. Gold standard Fourier shell correlation (FSC) curves with estimated resolutions.

d. Angular distribution of the VAR2CSA–Fcμ–J particles used in the final 3D reconstruction.

e. FSC curve for the structural model versus the cryo-EM map.

f. Compared to the VAR2CSA–CSA structure (PDB ID: 7JGH)[1], a swing of the DBL5ε–DBL6ε arm is observed. The density map of VAR2CSA in the VAR2CSA–Fcμ–J complex is shown as a light gray surface. The DBL3X and DBL5ε domains of VAR2CSA are colored as in Fig. 2, whereas the rest of the structurally modeled regions of VAR2CSA is shown in dark gray. VAR2CSA in the VAR2CSA–CSA complex is shown as pink ribbons for comparison, with CSA shown in spheres.

g. Density maps of representative regions at the VAR2CSA–Fcμ interface.

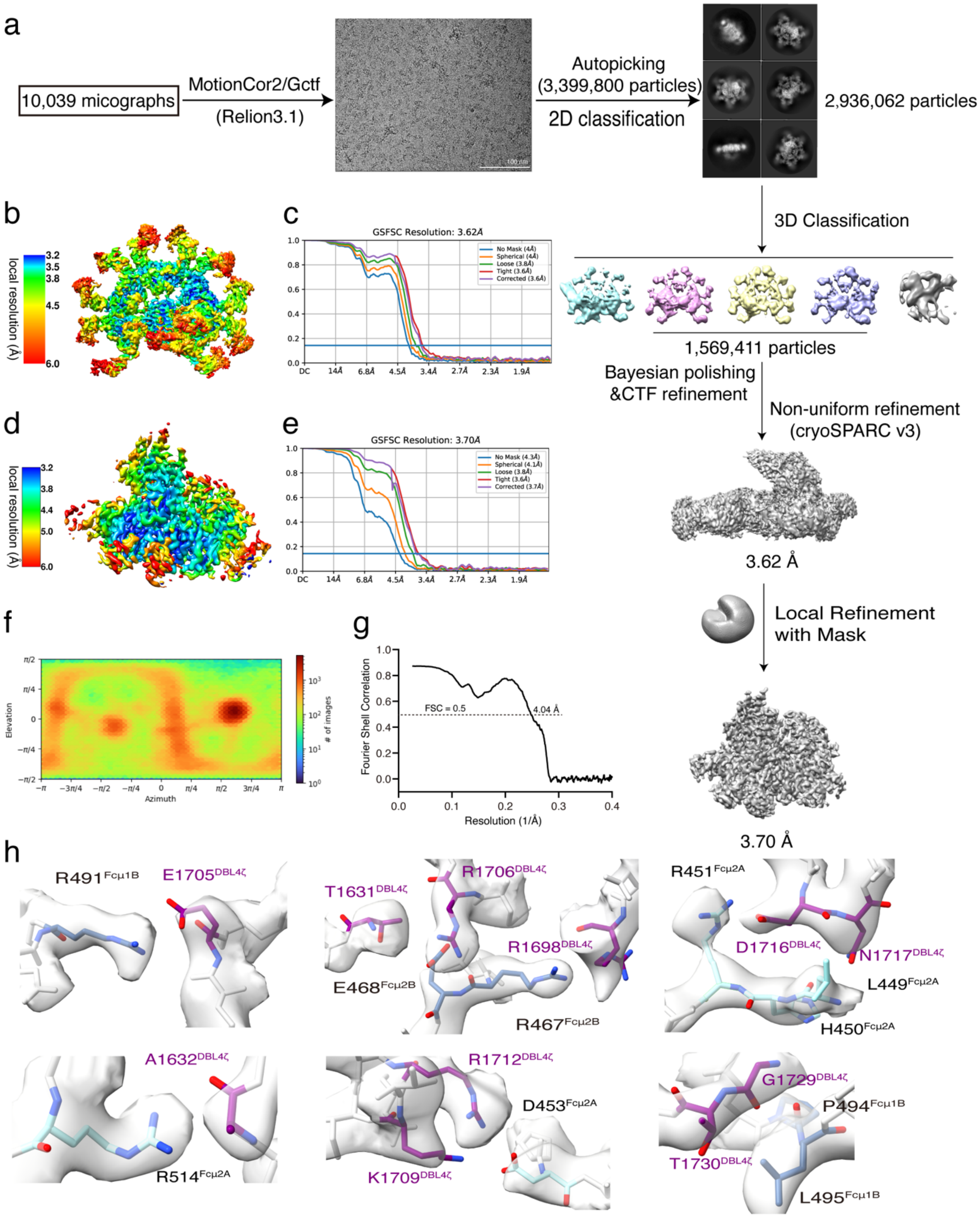

Supplementary Figure 3. Workflow for the 3D reconstruction of the TM284VAR1–Fcμ–J cryo-EM structure.

a. Flow chart for image processing of TM284VAR1–Fcμ–J.

b. Resolution estimations of the overall map of TM284VAR1–Fcμ–J.

c. FSC curves with estimated resolutions of TM284VAR1–Fcμ–J.

d. Resolution estimations of the local map around the binding interface between TM284VAR1 and Fcμ–J.

e. FSC curves with estimated resolutions for the local map.

f. Angular distribution of the TM284VAR1–Fcμ–J particles used in final 3D reconstruction.

g. FSC curve for the structural model versus the cryo-EM map.

h. Density maps of representative regions at the TM284VAR1–Fcμ interface.

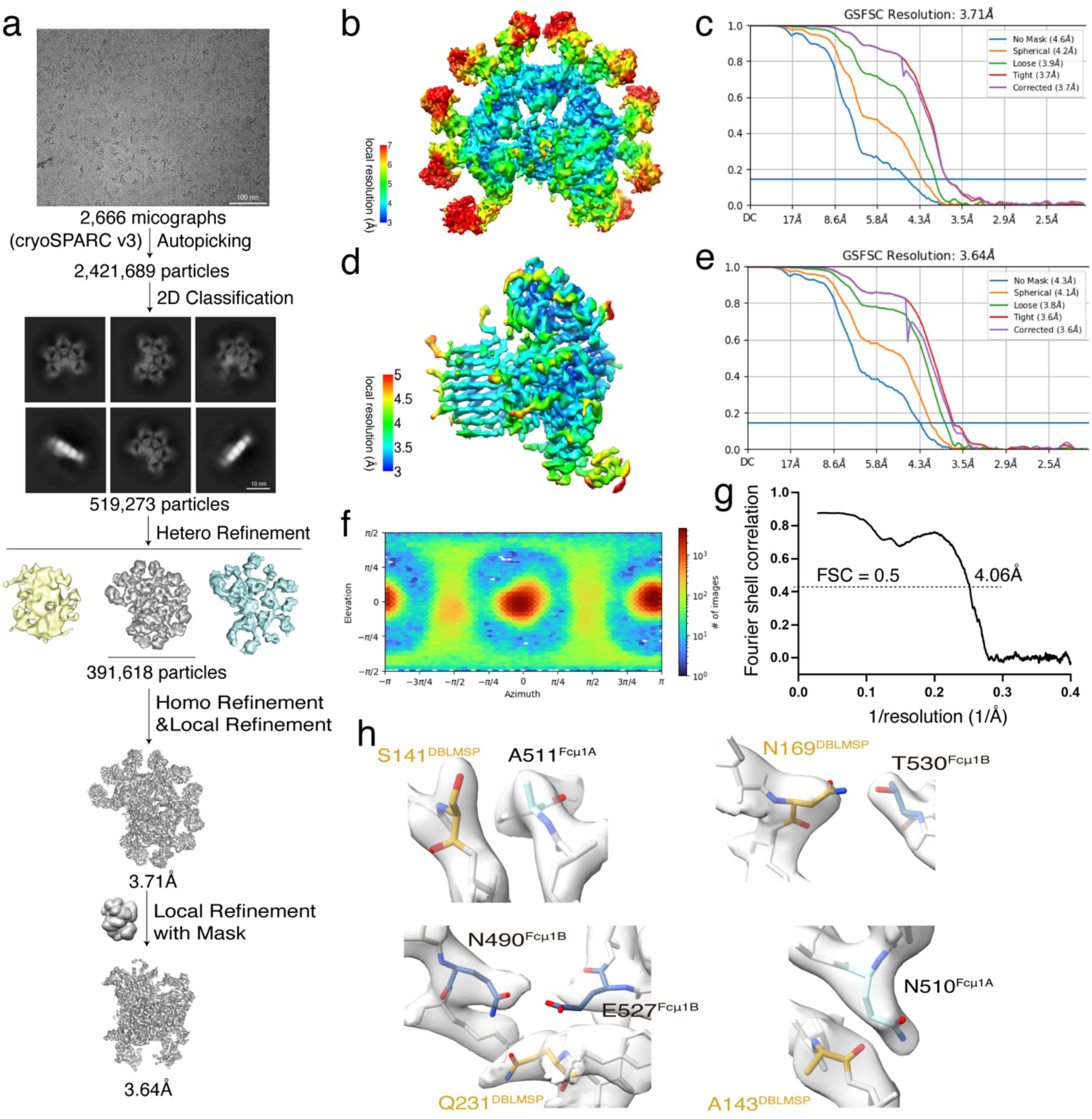

Supplementary Figure 4. Workflow for the 3D reconstruction of the DBLMSP$_{DBL}$–Fcμ–J cryo-EM structure.

a. Flow chart for image processing of DBLMSP$_{DBL}$–Fcμ–J.

b. Resolution estimations of the final map of DBLMSP$_{DBL}$–Fcμ–J.

c. FSC curves with estimated resolutions of DBLMSP$_{DBL}$–Fcμ–J.

d. Resolution estimations of the local map around the binding interface of DBLMSP$_{DBL}$–Fcμ–J.

e. FSC curves for the local map of DBLMSP$_{DBL}$–Fcμ–J.

f. Angular distribution of the DBLMSP$_{DBL}$–Fcμ–J particles used in final reconstruction.

g. FSC curve for the structural model versus the cryo-EM map.

h. Density maps of representative regions at the DBLMSP$_{DBL}$–Fcμ interface.

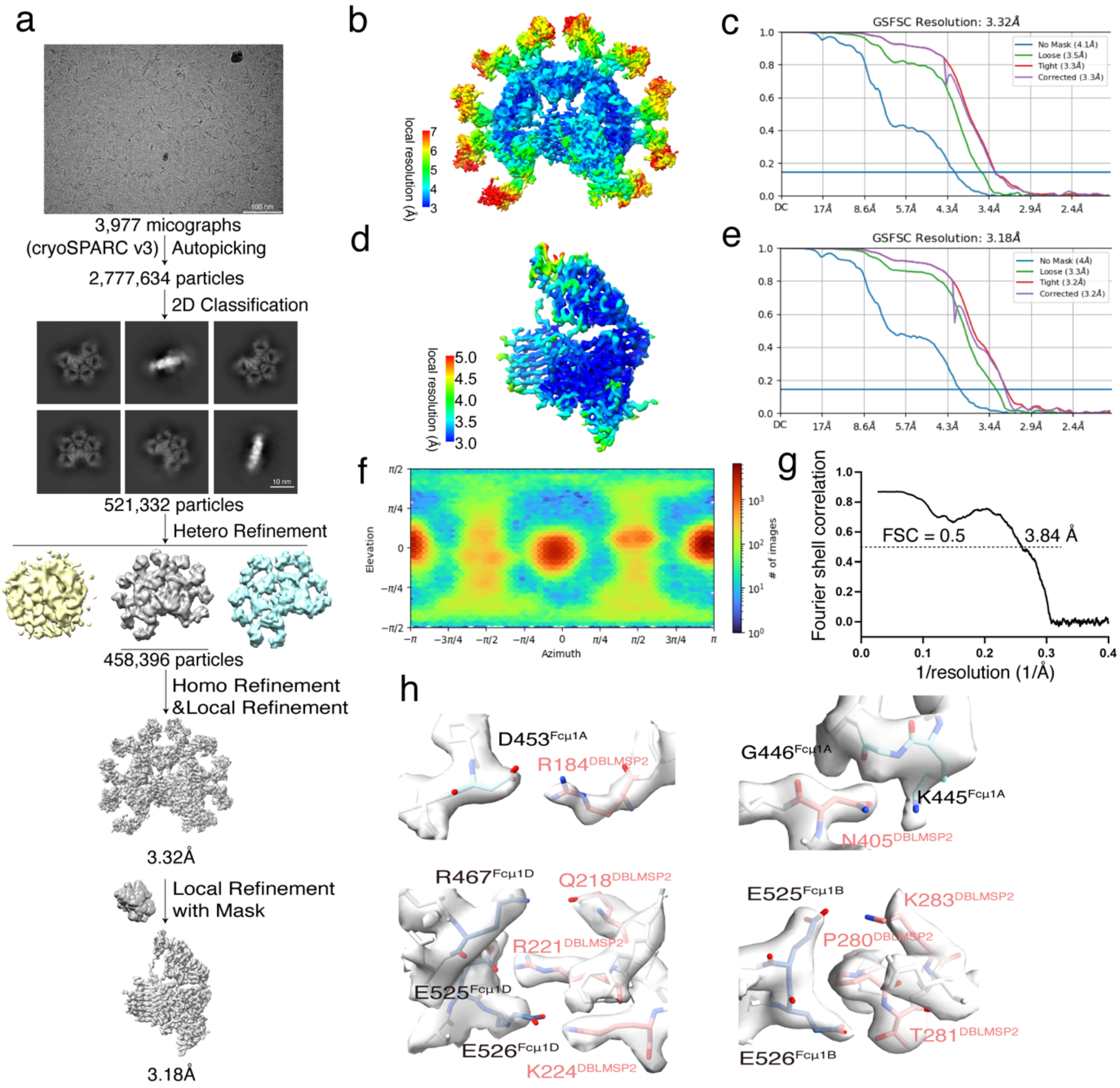

Supplementary Figure 5. Workflow for the 3D reconstruction of the DBLMSP2$_{DBL}$–Fcμ–J cryo-EM structure.

a. Flow chart for image processing of DBLMSP2$_{DBL}$–Fcμ–J.

b. Resolution estimations of the final map of DBLMSP2$_{DBL}$–Fcμ–J.

c. FSC curves with estimated resolutions of DBLMSP2$_{DBL}$–Fcμ–J.

d. Resolution estimations of the local map around the binding interface of DBLMSP2$_{DBL}$–Fcμ–J.

e. FSC curves for the local map of DBLMSP2$_{DBL}$–Fcμ–J.

f. Angular distribution of the DBLMSP2$_{DBL}$–Fcμ–J particles used in final reconstruction.

g. FSC curve for the structural model versus the cryo-EM map.

h. Density maps of representative regions at the DBLMSP2$_{DBL}$–Fcμ interface.

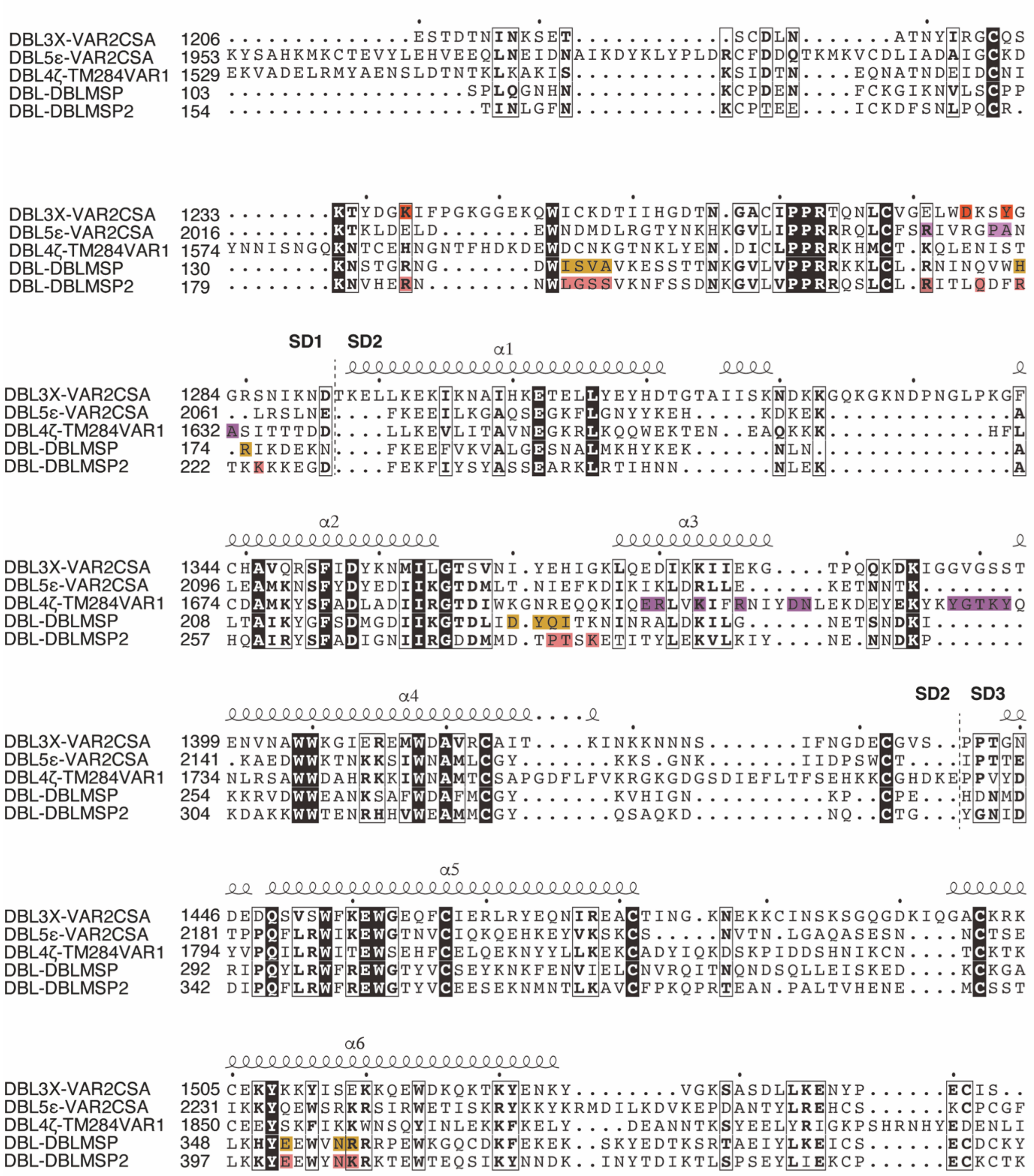

Supplementary Figure 6. Sequence alignment of VAR2CSA-DBL3X, VAR2CSA-DBL5ε, TM284VAR1-DBL4ζ, DBLMSP-DBL, and DBLMSP2-DBL.

Residues critically involved in binding to IgM in each DBL domain are highlighted. The multiple sequence alignment was performed using Clustal X[2]. This figure was prepared using ESPript 3.0 (https://espript.ibcp.fr)[3].

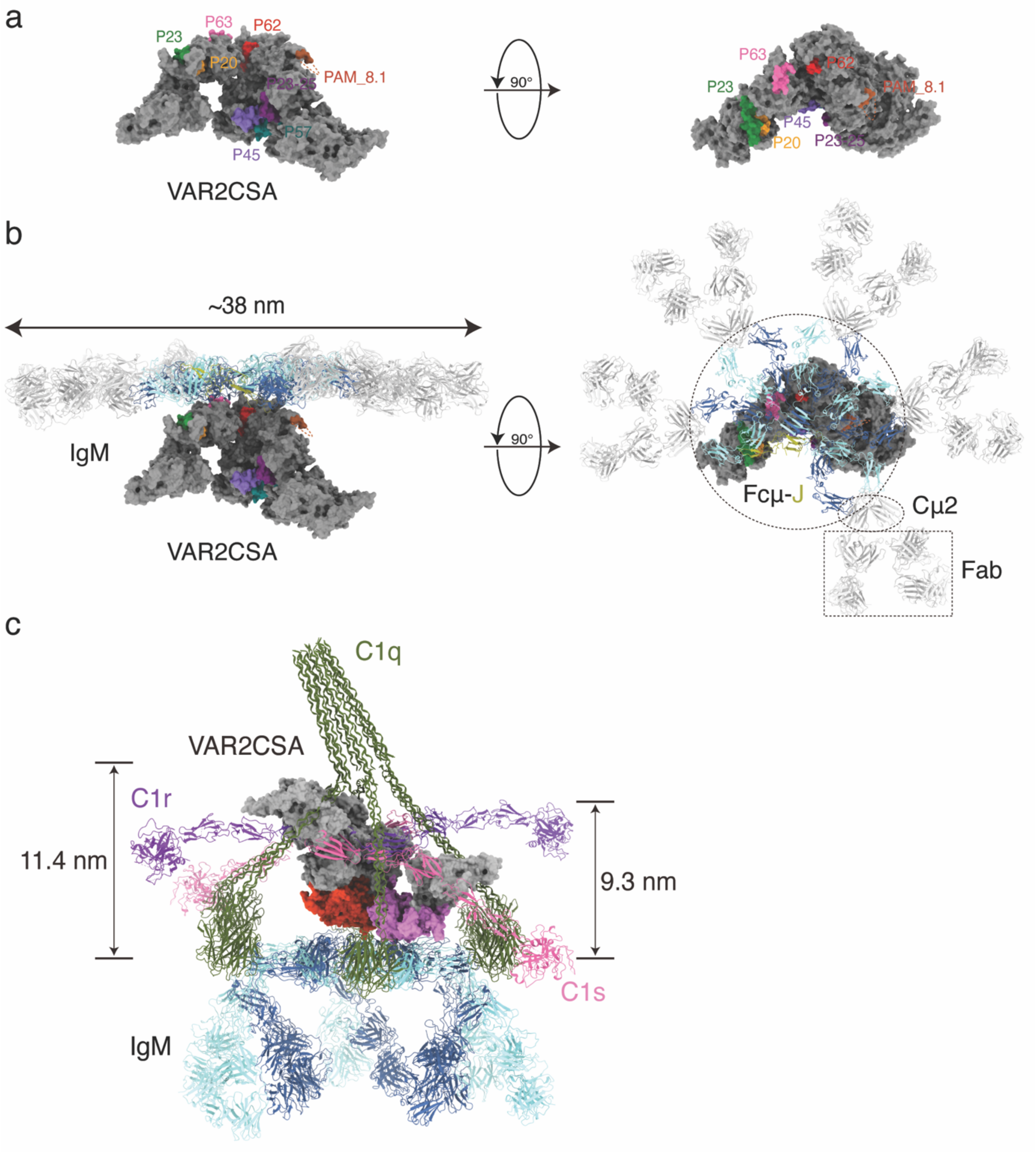

Supplementary Figure 7. VAR2CSA hijacks IgM to bury IgG epitopes and inhibit complement-dependent cytotoxicity.

a. Surface view of VAR2CSA, with the known IgG epitopes highlighted.

b. A model of VAR2CSA in complex with a full-length IgM molecule. This figure is generated by superimposing the VAR2CSA–Fcμ–J structure to the IgM structural model[4], with the antigen-binding fragments further putatively adjusted to fully-extended conformations.

c. A model of how VAR2CSA could inhibit complement-dependent cytotoxicity by preventing the interaction between IgM and C1. This figure is generated by superimposing the VAR2CSA–Fcμ–J structure to the IgM–C1 structural model[4]. VAR2CSA is shown in a surface representation, whereas the other proteins are shown in ribbons.

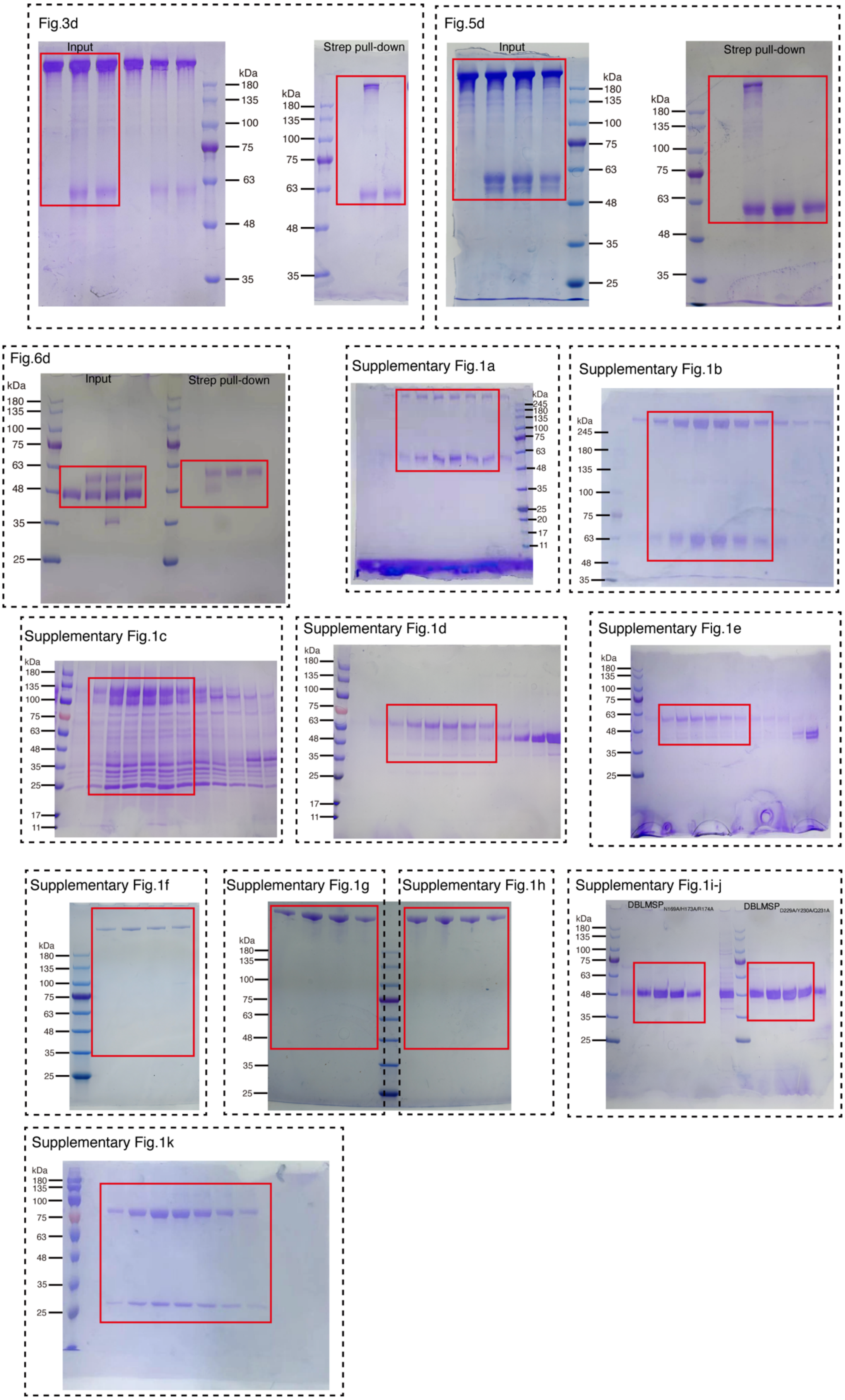

Supplementary Figure 8. Uncropped versions of gels presented in this study.

Supplementary Table 1. Cryo-EM data collection, refinement and validation statistics.

	VAR2CSA-Fcμ-J	TM284VAR-Fcμ-J	DBLMSP-Fcμ-J	DBLMSP2-Fcμ-J
**Data collection and processing**				
Voltage (kV)	300	300	300	300
Microscope	FEI Titan Krios G3	FEI Titan Krios G3	FEI Titan Krios G3i	FEI Titan Krios G3
Camera	K3 Summit (Gatan)	K2 Summit (Gatan)	K3 Summit (Gatan)	K3 Summit (Gatan)
Magnification (calibrated)	81,000×	165,000×	64,000×	81,000×
Electron exposure ($e^-/Å^2$)	60	59.74	50	60
Exposure rate ($e^-/Å^2/s$)	18.75	11.688	19.76	21.47
Number of frames collected per micrograph	32	32	32	40
Energy filter slit width	20 eV	20 eV	20 eV	20 eV
Automation software	EPU	SerialEM	EPU	EPU
Defocus range (μm)	-1.0 to -2.0	-1.0 to -2.0	-1.0 to -1.5	-1.1 to -1.5
Pixel size (Å)	1.07	0.828	1.08	1.07
Micrographs used	7,103	10,039	2,666	3977
Symmetry imposed	C1	C1	C1	C1
Initial particle images	3,477,418	3,399,800	2,421,689	2777634
Final particle images	690,345	849,826	391,618	458396
Overall map resolution at 0.143 FSC of masked reconstruction (Å)	3.56	3.62	3.71	3.32
Local map resolution at 0.143 FSC of masked reconstruction (Å)	/	3.7	3.64	3.18
Overall map sharpening B factor ($Å^2$)	-138.3	-163.7	-105.7	-90.6
Local map sharpening B factor ($Å^2$)	/	-124.7	-116.5	-89.5
**Refinement**				
Refinement package	Phenix v1.19	Phenix v1.19	Phenix v1.19	Phenix v1.19
Map-model CC				
CC_mask	0.74	0.73	0.78	0.68
CC_box	0.7	0.7	0.77	0.68
CC_peaks	0.6	0.59	0.68	0.59
CC_volume	0.72	0.7	0.76	0.66
Model composition				
Non-hydrogen atoms	26,471	20,717	20,817	20783
Protein residues	3,336	2,616	2,636	2630
Ligands	12	12	12	12
Bond lengths (Å)	0.003	0.003	0.003	0.003
Bond angles (°)	0.603	0.658	0.668	0.601
B factors ($Å^2$)				
Protein	104.17	114.42	122.31	91.24
Ligands	70.7	84.02	93.47	70.59
Validation				
MolProbity score	1.67	1.78	1.73	1.59
Clashscore	6.19	7.32	8.71	6.6
Poor rotamers (%)	0	0	0.13	0
Ramachandran plot				
Favored (%)	95.29	94.56	96.12	96.49
Allowed (%)	4.71	5.44	3.88	3.51
Disallowed (%)	0	0	0	0
Cβ outliers (%)	0	0	0	0
CaBLAM outliers (%)	2.37	3.01	2.15	1.65

Supplementary References

1. Ma, R. *et al*. Structural basis for placental malaria mediated by Plasmodium falciparum VAR2CSA. *Nat Microbiol* **6**, 380-391, doi:10.1038/s41564-020-00858-9 (2021).
2. Larkin, M. A. *et al*. Clustal W and Clustal X version 2.0. *Bioinformatics* **23**, 2947-2948, doi:10.1093/bioinformatics/btm404 (2007).
3. Robert, X. & Gouet, P. Deciphering key features in protein structures with the new ENDscript server. *Nucleic Acids Res* **42**, W320-324, doi:10.1093/nar/gku316 (2014).
4. Sharp, T. H. *et al*. Insights into IgM-mediated complement activation based on in situ structures of IgM-C1-C4b. *Proc Natl Acad Sci U S A* **116**, 11900-11905, doi:10.1073/pnas.1901841116 (2019).
