# Supplementary material for: Plasmodium falciparum has evolved multiple mechanisms to hijack human immunoglobulin M
Source: Nat Commun. 2023 May 8;14:2650. doi: 10.1038/s41467-023-38320-z (PMC10167334; doi:10.1038/s41467-023-38320-z)
Supplement: Supplementary file 3 — Reporting Summary [file 41467_2023_38320_MOESM3_ESM.pdf]

nature portfolio

Corresponding author(s): Junyu Xiao
Last updated by author(s): Apr 8, 2023

Reporting Summary

Nature Portfolio wishes to improve the reproducibility of the work that we publish. This form provides structure for consistency and transparency in reporting. For further information on Nature Portfolio policies, see our Editorial Policies and the Editorial Policy Checklist.

Statistics

For all statistical analyses, confirm that the following items are present in the figure legend, table legend, main text, or Methods section.

n/a	Confirmed	
☐	☒	The exact sample size (*n*) for each experimental group/condition, given as a discrete number and unit of measurement
☐	☒	A statement on whether measurements were taken from distinct samples or whether the same sample was measured repeatedly
☒	☐	The statistical test(s) used AND whether they are one- or two-sided<br>*Only common tests should be described solely by name; describe more complex techniques in the Methods section.*
☒	☐	A description of all covariates tested
☒	☐	A description of any assumptions or corrections, such as tests of normality and adjustment for multiple comparisons
☐	☒	A full description of the statistical parameters including central tendency (e.g. means) or other basic estimates (e.g. regression coefficient) AND variation (e.g. standard deviation) or associated estimates of uncertainty (e.g. confidence intervals)
☒	☐	For null hypothesis testing, the test statistic (e.g. *F*, *t*, *r*) with confidence intervals, effect sizes, degrees of freedom and *P* value noted<br>*Give P values as exact values whenever suitable.*
☒	☐	For Bayesian analysis, information on the choice of priors and Markov chain Monte Carlo settings
☒	☐	For hierarchical and complex designs, identification of the appropriate level for tests and full reporting of outcomes
☒	☐	Estimates of effect sizes (e.g. Cohen's *d*, Pearson's *r*), indicating how they were calculated

Our web collection on statistics for biologists contains articles on many of the points above.

Software and code

Policy information about availability of computer code

Data collection	Serial EM v3.8.7 and EPU v2.12 were used to collect the cryo-EM data.
Data analysis	MotionCor2 v1.4.4, Gctf v1.06, cryoSPARC v3.2 and RELION v3.1 were used to process the cryo-EM data.<br>The local resolution map was analyzed using ResMap and displayed using UCSF ChimeraX v1.4.<br>Model was docked into map using UCSF Chimera v1.15.<br>Structural modeling and refinement were performed using Coot v0.9.4.1 and Phenix v1.19.<br>The complement dependent cytotoxicity assay data were analyzed with GraphPad Prism (v8.0.2)<br>The SPR data were analyzed with Biacore Evaluation Software v2.0.<br>The sequence alignment was analyzed with Clustal X v2.1.

For manuscripts utilizing custom algorithms or software that are central to the research but not yet described in published literature, software must be made available to editors and reviewers. We strongly encourage code deposition in a community repository (e.g. GitHub). See the Nature Portfolio guidelines for submitting code & software for further information.

Data

Policy information about availability of data

All manuscripts must include a data availability statement. This statement should provide the following information, where applicable:

- Accession codes, unique identifiers, or web links for publicly available datasets
- A description of any restrictions on data availability
- For clinical datasets or third party data, please ensure that the statement adheres to our policy

Cryo-EM density maps of VAR2CSA-Fcμ-J, TM284VAR1-Fcμ-J, DBLMSP-Fcμ-J, and DBLMSP2-Fcμ-J have been deposited in the Electron Microscopy Data Bank with accession codes EMD-33542, EMD-33547 (EMD-33548 for the local map), EMD-33538 (EMD-33539 for the local map), and EMD-33805 (EMD-33806 for the local map), respectively. Structural coordinates have been deposited in the Protein Data Bank with accession codes 7Y0H, 7Y0J, 7Y09, and 7YG2. Previous published structural coordinates used in this study include 6KXS, 3CML, 7JGE, and 7JGF.

Human research participants

Policy information about studies involving human research participants and Sex and Gender in Research.

Reporting on sex and gender	N/A
Population characteristics	N/A
Recruitment	N/A
Ethics oversight	N/A

Note that full information on the approval of the study protocol must also be provided in the manuscript.

Field-specific reporting

Please select the one below that is the best fit for your research. If you are not sure, read the appropriate sections before making your selection.

☒ Life sciences ☐ Behavioural & social sciences ☐ Ecological, evolutionary & environmental sciences

For a reference copy of the document with all sections, see nature.com/documents/nr-reporting-summary-flat.pdf

Life sciences study design

All studies must disclose on these points even when the disclosure is negative.

Sample size	The number of particles used for EM reconstructions has been stated in the methods section. No statistical method has been used to predetermine sample size.
Data exclusions	Particle exclusion in cryoSPARC or Relion has been performed followed standard procedures built in these programs. No other data was excluded.
Replication	Three replications for SPR experiments and pull down assays have been performed including those mentioned in the manuscript. The CDC assays have been repeated simultaneously two times in an experiment and three such replications were carried out independently. All attempts at replication were successful.
Randomization	Not applicable since no groups to be allocated.
Blinding	Not applicable since no groups to be allocated.

Reporting for specific materials, systems and methods

We require information from authors about some types of materials, experimental systems and methods used in many studies. Here, indicate whether each material, system or method listed is relevant to your study. If you are not sure if a list item applies to your research, read the appropriate section before selecting a response.

Materials & experimental systems

n/a	Involved in the study
☐	☒ Antibodies
☐	☒ Eukaryotic cell lines
☒	☐ Palaeontology and archaeology
☒	☐ Animals and other organisms
☒	☐ Clinical data
☒	☐ Dual use research of concern

Methods

n/a	Involved in the study
☒	☐ ChIP-seq
☒	☐ Flow cytometry
☒	☐ MRI-based neuroimaging

Antibodies

Antibodies used

Anti-CD20 and anti-RBD IgM antibodies are produced in this study, as described in the methods section. The dilution used for each antibody is also specified in the methods section of the manuscript.

Validation

The functions of these antibodies have been validated by the complement-dependent cytotoxicity assay shown in Figure 3e and Supplementary figure 1l.

Eukaryotic cell lines

Policy information about cell lines and Sex and Gender in Research

Cell line source(s)

Sf21 insect cells, High Five insect cells, and HEK293F cells were originally purchased from ATCC. OCI-Ly10 cell, originally purchased from ATCC, was kindly provided by Prof. Xiaoguang Lei (Peking University).

Authentication

The cell lines were not authenticated.

Mycoplasma contamination

The cells were free of Mycoplasma contamination as tested by standard PCR method.

Commonly misidentified lines
(See ICLAC register)

No commonly misidentified lines were used.
